# Fungal-Derived Biobased Filaments for Textile Applications: Effect of Plasticizer and Cross-Linking Agent

**DOI:** 10.1021/acsomega.6c05677

**Published:** 2026-10-19

**Authors:** Hui Deng, Maria Persson, E. R. Kanishka B. Wijayarathna, Fathi Gouda, Göksu Cinar Ciftci, Akram Zamani

**Affiliations:** † Department of Textile Technology, Faculty of Textiles, Engineering and Business, 1802 University of Borås, SE-501 90 Borås, Sweden; ‡ Swedish Centre for Resource Recovery, Faculty of Textiles, Engineering and Business University of Borås, Borås SE-501 90, Sweden; § Department of Engineering, Faculty of Textiles, Engineering and Business University of Borås, Borås SE-501 90, Sweden; ∥ Bioeconomy and Health Division, RISE Research Institutes of Sweden, Stockholm SE-11428, Sweden

## Abstract

Fungal cell wall polymers derived from food waste have recently emerged as a renewable platform for novel biobased fibers. In this study, fungal biomass of *Rhizopus delemar* cultivated on bread waste was processed to isolate the alkali-insoluble cell wall material rich in chitin and chitosan, which was converted into a hydrogel spinning dope for dry gel spinning of continuous filaments. A molecular design strategy was employed to tailor filament performance using vanillin as a biobased cross-linker and glycerol as a plasticizer. The impact of these modifications on the properties of the filaments was systematically evaluated. Vanillin provided only marginal improvement in tenacity, suggesting that its incorporation did not substantially reinforce the chitosan-rich network. In contrast, glycerol incorporation significantly enhanced filament ductility and reduced brittleness during postspinning deformation processes such as twisting, thereby enabling stable yarn formation, although with a reduction in tensile strength. Multifilament yarns were successfully fabricated, and knitted textile specimens were produced for the first time from fungal-derived yarns, demonstrating structural integrity under textile processing conditions. This study establishes a structure–property framework for balancing stiffness and flexibility in fungal-based filaments and advances their development toward engineered, renewable textile materials.

## Graphical abstract

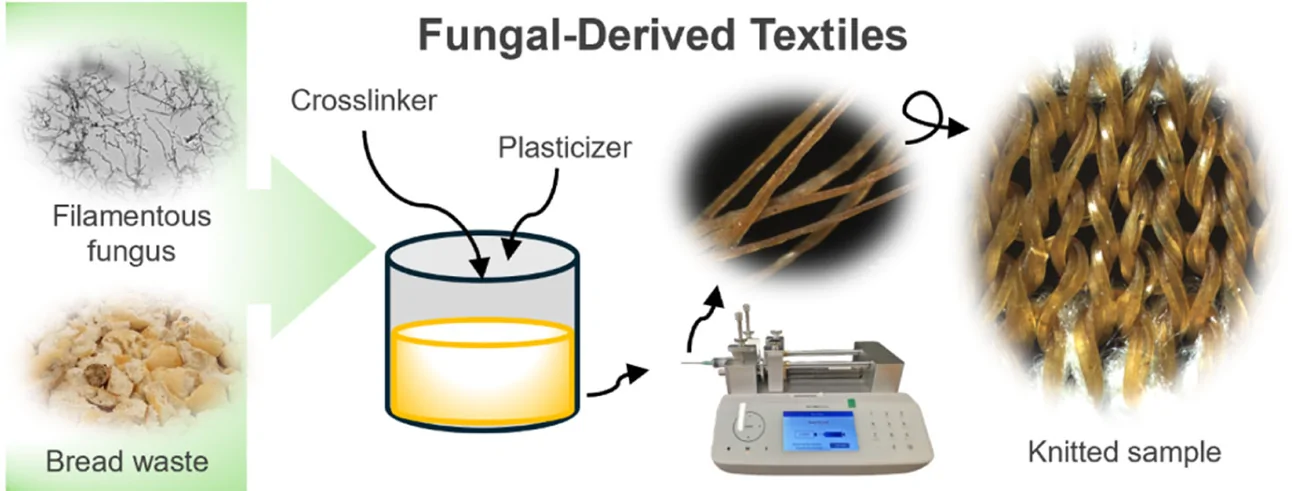

## 1 Introduction

Food waste and textile pollution are two pressing global sustainability challenges. Bakery waste constitutes a significant loss throughout all stages of the food supply chain, from production to consumption. For instance, at the retail level alone, bakery products can account for approximately 15–30% of total food waste, underscoring both the economic and environmental relevance of this waste stream.^1^ At the same time, the fashion industry faces criticism for its heavy reliance on synthetic fibers, which shed microplastics and persist in the environment due to poor biodegradability.^2^ Developing renewable, biodegradable textile fibers from food waste therefore offers an opportunity to address both problems simultaneously.

In recent years, research efforts have increasingly focused on valorizing food waste as a substrate for microbial cultivation for biopolymer synthesis and fiber production. For example, food waste has been employed to generate microbial polyester, such as polyhydroxyalkanoates (PHAs),^3^ which can be spun into textile fibers.^4^ Similarly, bacterial cellulose produced from food waste streams^5^ has shown potential in fiber development.^6^ Recently, we demonstrated the feasibility of spinning filaments from the cell walls of filamentous fungi cultivated on food waste,^7^ further highlighting the potential of this circular approach.

Food waste can serve as an effective substrate for cultivating filamentous fungi such as *Rhizopus delemar*, providing carbohydrates, proteins, and minerals required for growth.^8^ This fungus belongs to the class of *mucoromycetes*, the filamentous fungi which synthesize both chitin and chitosan in their cell wall. While chitosan can be isolated from cell wall, our previous research has shown the entire fungal cell wall material can be directly processed into a hydrogel and spun into filaments, avoiding conventional chitosan isolation and improving material efficiency and yield.^9^ Filament production has been demonstrated using both wet spinning^10^ and dry-gel spinning methods.^11^ Dry-gel spinning of fungal monofilaments, recently developed by our group, eliminates the need for a coagulation stage, enabling formation of continuous monofilaments.^11^

These studies demonstrate that food waste as an abundant resource can be repurposed to develop novel, biobased filaments derived from filamentous fungi. However, the performance of fungal filaments as textile materials remains largely underexplored. Our preliminary experiments indicated that filaments produced by dry-gel spinning are relatively brittle, making their processing in conventional textile operations, such as knitting, challenging. Although chitin- and chitosan-based fibers derived from crustacean shells are well-known, it remains unclear how cross-linkers and plasticizers can be used to improve their mechanical properties. In contrast, studies on chitosan films have shown that cross-linking and plasticization can substantially enhance mechanical performance and flexibility.^12,13^ While aldehyde-based agents such as glutaraldehyde are effective, they present toxicity concerns.^14^ Vanillin, a natural aromatic compound, has recently gained attention for improving the physical and mechanical properties of chitosan-based films while also contributing a pleasant scent.^15,16^ However, the effects of cross-linking on fungal-derived chitosan and, specifically, on the mechanical properties of fungal filaments remain unknown.

The objective of this study was to systematically evaluate the performance of fungal filaments as a textile material and to design strategies for improving the mechanical properties and processability. We hypothesized that use of a cross-linking agent and plasticizer would enable fungal filaments to achieve the mechanical performance and handling characteristics required for integration into conventional textile processing. Specifically, we investigated the use of vanillin as a biobased cross-linking agent to enhance filament strength and glycerol as a plasticizer to increase flexibility and facilitate textile formation.

## 2 Materials and Methods

### 2.1 Chemicals and Materials

*R. delemar* CBS145940 (Westerdijk Fungal Biodiversity Institute, Netherlands) originally isolated from tempeh was used. Ethanol (absolute) was purchased from VWR. Lactic acid (85%), yeast extract, α-amylase from *Bacillus licheniformis* (heat-stable), vanillin (99%), sodium hydroxide (98%), and glycerol (≥99%) were all supplied from Sigma-Aldrich. Waste bread (unsold) was obtained from ICA city Borås, Sweden. The bread was manually broken into smaller pieces and dried at room temperature, then milled into a powder with a particle size of <3 mm.

### 2.2 Fungal Cultivation

Fungal cultivation was done according to our previous study^11^ and is briefly explained below. All cultivations were carried out using a substrate composed of the liquid fraction from bread hydrolysis (with α-amylase), adjusted to 4% total solids and supplemented with 1 g/L yeast extract. Fungal cultivation was done in a three-step scale-up process (i.e., 500 mL Erlenmeyer flasks, 26 L bubble column bioreactor, and 1300 L bubble column bioreactor). An inoculum volume of 2% was maintained in all the cultivation steps.

In the first step, two 500 mL Erlenmeyer flasks with 200 mL of substrate were inoculated with 8 mL of spore suspension. The flasks were incubated at 35 °C for 24 h in a water bath shaker (Grant Instruments Ltd., UK) at 110 rpm. The second cultivation step was done in 26 L bubble column reactor with 20 L of substrate. The bioreactor was sterilized in situ, and the substrate was sterilized using an autoclave (VX95, Systec, Germany). The entire cultivation broth from the Erlenmeyer flasks was used to inoculate the 26 L bioreactor. Cultivation proceeded for 24 h at 35 °C and 1 VVM (volume of air per volume of liquid per minute) aeration.

In the final scale-up step, a 1300 L pilot scale bubble column reactor containing 1000 L of substrate was used. The empty reactor was sterilized once, and the reactor filled with substrate was sterilized again prior to the inoculation. The 20 L cultivation broth obtained from the 26 L bubble column bioreactor was used as the inoculum. This last cultivation was carried out for 48 h at 35 °C and 0.5 VVM aeration.

After cultivation, the biomass was harvested, washed twice with water to remove any unconsumed substrate, and packed in ziplock bags and stored in freezer until use.

### 2.3 Preparation of Alkali Insoluble Material

Alkali insoluble material (AIM) was prepared according to our previous study.^11^ A 1% (w/v) suspension of fungal biomass was prepared in water and processed using an ultrafine grinder (Masuko, Japan) equipped with fine silicon carbide grinding stones (MKE #46) set at an open gap of 50 μm. The grinding was done in two stages: first pass at low speed (750 rpm) followed by two more passes at high speed (2700 rpm). The resulting slurry was filtered through 210 μm filter bags (Brew Bag, USA).

The filtered biomass was subsequently mixed with a 0.2 M NaOH solution (50 mL per gram of dry biomass) and stirred thoroughly until the suspension became homogeneous. The alkali treatment was conducted in an autoclave (VX95, Systec, Germany) at 120 °C for 20 min. After that heat treatment, the AIM was separated by filtration using the same 210 μm filter bag and washed repeatedly with water until the filtrate reached a neutral pH (6–7), as confirmed by pH-indicator paper.

### 2.4 Preparation of Fungal Hydrogel

Lactic acid (0.25 M) was added to AIM at a ratio of 5 mL per 0.1 g dry weight of AIM, resulting in a hydrogel. To adjust the pH to 3, sodium hydroxide (0.2 M) and lactic acid (0.25 M) were used. The fungal hydrogel at pH 3 was ground using an ultrafine grinder for six cycles with an open gap of 50 μm at a speed of 2700 rpm. Afterward, the ground hydrogel was vacuum filtered using a vacuum funnel set up (Merck, Germany) through a nylon filter of 30 μm (Spectra/Mesh, Woven Filters) to obtain hydrogels with target concentration (5.5 wt %).

### 2.5 Dry Gel Spinning of Fungal Hydrogel

Filament spinning was performed using a high-pressure syringe pump (Fusion 6000X, CHEMYX, USA), which provided a stable and continuous flow, enabling the production of filaments up to 10 m in length. According to a preliminary study, a hydrogel concentration of 5.5 wt % was identified as optimal for stable filament spinning. The hydrogel was carefully loaded into a syringe to minimize air entrapment, as trapped air can disrupt extrusion and hinder the formation of continuous filaments. The syringe was mounted on the pump, and a stainless-steel needle was positioned in front of a rotating collector wheel wrapped with Parafilm (Bemis, USA). The hydrogel was extruded through a 1.2 mm or 0.8 mm diameter needle at a controlled rate ranging from 0.25 to 1 mL/min, while the collector rotated at an adjustable speed (50–210 rpm) synchronized with the extrusion rate to ensure smooth winding of the of the as-spun monofilament without breakage. The resulting monofilament was then left to dry at room temperature on the Parafilm surface.

#### 2.5.1 Spinning of Filaments Containing Different Vanillin Concentrations

To incorporate vanillin as a cross-linking agent, a stock solution was prepared by dissolving 2 g of vanillin in 10 mL ethanol. The required volume of this solution was added to hydrogels with a fixed gel concentration of 5.5 wt % to obtain formulations containing 0%, 20%, 40%, 60%, 80%, and 100 wt % vanillin. The vanillin concentration was expressed as the percentage of vanillin weight relative to the hydrogel’s dry weight. Prior to spinning, preliminary tests were conducted to establish suitable drying conditions for improving the vanillin-assisted cross-linking reaction. Accordingly, the effect of drying conditions on monofilament properties was investigated using a formulation containing 30 wt % vanillin. The monofilaments were dried under three different conditions: air-dried at room temperature, oven-dried at 60 °C for 1 h, and air-dried at room temperature followed by annealing at 60 °C for 1 h.

#### 2.5.2 Incorporation of Glycerol as Plasticizer

Hydrogels (5.5 wt %) were prepared with a fixed vanillin concentration of 20 wt % and glycerol concentrations of 0, 5, 10, 15, and 20 wt %. For comparison, control hydrogels of the same concentration (5.5 wt %) without vanillin and containing 0, 5, and 20 wt % glycerol were also prepared. After thorough mixing, monofilaments were spun following the procedure described in Section 2.5.

### 2.6 Twisting of Monofilaments

Yarns composed of three to six monofilaments were twisted at 16–20 turns per meter (TPM) using a yarn twist tester (Max Kohl A.G., Chemnitz, Germany). Prior to twisting, the monofilaments were heat-steamed for approximately 30 s to moisten the filaments and increase their flexibility. After twisting, the plied yarns were further heat-steamed for 2 min while positioned on the twist tester to promote adhesion and allow drying.^11^

### 2.7 Knitting

A single jersey weft structure was adopted for the knitting process. The fabrics were knitted in intarsia mode using a Protti Intarsia hand-knitting machine (gauge 7, stitch size 14) equipped with 10–20 needles. A lightweight comb was used to ensure smooth loop formation and fabric stability. Yarns were manually fed from right to left, and a tension control device ensured stable yarn delivery. The final row was secured by manually casting off the loops using a latch tool to prevent unraveling.

### 2.8 Characterizations

#### 2.8.1 Microscopy Analysis

Images of the monofilaments, twisted monofilament yarns, and knitted fabric were captured using a SMZ-800 model stereoscopic microscope (Nikon Instruments, Japan), with a zoom range 1.0×–6.3× (a total magnification of 10×–63×).

To conduct scanning electron microscopy (SEM) analysis, the knitted samples were mounted on a sample holder using carbon tape and then trimmed around the edge of the holder. The monofilaments were placed between two pieces of carbon tape, with the release paper still attached on the outside. The monofilaments were cut with sharp scissors, and the cut sections were placed on another piece of carbon tape for top-surface imaging. SEM imaging was performed in high vacuum (HV) mode at 2 kV using a FEI, Quanta 250 FEG environmental SEM (Thermo Fisher Scientific). First, all top surfaces were imaged, followed by the cross sections of the monofilaments.

The optical anisotropy and molecular orientation of neat (0 wt % glycerol, 0 wt% vanillin) and plasticized (20 wt % glycerol, 0 wt% vanillin) fungal monofilaments were evaluated using a Nikon Eclipse LV100ND microscope (Nikon Instruments, Japan) equipped with a high-resolution DS-Fi3 digital camera. The polarized light microscope operated in transmission mode with crossed polarizers. Individual filaments were mounted free-standing across an open cardboard frame using adhesive tape at both ends, allowing the fibers to be analyzed suspended in air without an underlying glass substrate to avoid parasitic background birefringence. The specimen frame was positioned on a graduated rotating stage. Micrographs were acquired at 50× magnification at targeted orientations relative to the polarizer axis, namely at the maximum transmission angle (45°) and the extinction angle (0°). Image recording and dimensional calibrations were conducted using NIS-Elements software, and calibrated scale bars were applied directly to the images.

#### 2.8.2 Linear Density and Tensile Properties

The monofilaments were conditioned for 24 h at 20 ± 2 °C and 65 ± 2% relative humidity prior to testing. The linear density (tex, i.e., 1 tex = 1 g/1000 m) of the monofilaments was determined by weighing a 1 m length of each filament. This value was then used to estimate the appropriate pretension for mechanical testing. A pretension of 1 cN/tex was applied to filaments spun using needle with diameter of 1.2 mm, whereas a pretension of 0.3 cN/tex was applied to monofilaments spun through needle with 0.8 mm diameter. The gauge length and test speed were set to 20 mm and 20 mm/min, respectively. Tenacity and elongation at break were then measured using the Favimat + single-fiber tester (Textechno, Germany) equipped with standard clamps.

#### 2.8.3 Fourier Transform Infrared Spectroscopy

FTIR was performed to analyze the functional groups present in the monofilaments and their cross-linking with vanillin. Fungal filaments containing varying concentrations of vanillin were ground into a fine powder using a ball mill (Retsch, Germany) for 2 min at 30 Hz with three steel balls and the collected powder was subjected to FTIR analysis. FTIR spectra were recorded with a Nicolet iS10 FTIR spectrometer (Thermo Fisher Scientific Inc., Madison) with the Zn/Se crystal attenuated total reflectance (ATR) mode. At least 32 scans with a spectral resolution of 4 cm^–1^ were averaged in the range of 4000–400 cm^–1^. The results were analyzed using the software OriginPro 8.6 (OriginLab Corporation, Northampton Corporation, Northampton). The obtained spectra were normalized to the highest peak of each spectrum, and the resulting normalized spectra were used to analyze changes in chemical functionalities.

#### 2.8.4 Swelling Behavior

The swelling behavior of monofilaments was evaluated using samples containing varying amounts of vanillin (0, 20, and 80 wt %) without plasticizer, in order to investigate the cross-linking effect of vanillin. Each sample was first weighed in its dry state, and the mass was recorded as ***M***_**0**_. The monofilaments were then immersed in distilled water at room temperature for 2 h. After immersion, the samples were carefully removed and gently blotted with filter paper to remove excess surface water, after which the swollen mass (***M***_**s**_) was recorded. The swelling ratio (***S***) was calculated according to the following equation

$$S=\frac{M_{s}-M_{0}}{M_{0}}$$

Three samples of each monofilament were tested, and the mean swelling ratio was reported.

### 2.9 Filament Mechanical Property Comparison with Ashby’s Plots

The mechanical properties of the produced monofilaments were compared with commercially available textile fibers using Ashby’s plots with the help of Granta EduPack 2025 R1 (version 25.1.2, Ansys Inc., USA). The comparison was performed using the level 3 advanced material database, where specific strength was plotted against elongation at break. The specific strength of fungal monofilaments was calculated via the following equation using tenacity data.

Specificstrength(kNm/kg)=10×tenacity(cN/tex)

### 2.10 Statistical Analysis

Statistical analysis of the data was conducted using Minitab Statistical Software [version 22.2.2 (64 bit)]. Normality of the data was first assessed using the Anderson–Darling test. One-way analysis of variance (ANOVA) was then performed at a 95% confidence level to determine statistically significant differences (*p* < 0.05), followed by Tukey’s honestly significant difference (HSD) test for posthoc comparisons. When the data did not meet normality assumptions, a nonparametric Kruskal–Wallis ANOVA was applied. However, the power of this analysis is limited given the small number of replicates.

## 3 Results and Discussion

The increasing demand for sustainable textile materials has driven the exploration of biobased alternatives to replace conventional fossil-derived fibers. Herein fungal filaments were developed from a filamentous fungus (*R. delemar*) cultivated on food waste, offering a circular approach to textile production. Cell wall material extracted from the fungal biomass was processed into a hydrogel and spun into monofilaments using a dry gel spinning method. Effects of vanillin as cross-linking agent and glycerol as plasticizer on performance of the filaments as textile materials were investigated. The best resulting monofilaments were twisted into multifilament yarns and successfully knitted into fabric, demonstrating the potential of fungal-derived filaments for textile applications.

### 3.1 Preparation of Fungal Hydrogel and Dry Gel Spinning

Fungal hydrogels were prepared from AIM by protonating the amino groups of chitosan present in the fungal cell wall using lactic acid.^17^ The fungal biomass was subjected to alkaline treatment in an autoclave, resulting in AIM with a dark brown appearance (Figure 1a). After repeated washing with water until neutral pH, the AIM became light yellow (Figure 1b). Upon the addition of lactic acid (Figure 1c), gelling took place, and the resulting concentrated hydrogel exhibited a light-yellow color (Figure 1d). After adding vanillin, the hydrogel developed a distinct aromatic odor and had a more cohesive consistency during manual handling. In contrast, the addition of glycerol resulted in no noticeable changes in color or smell of the hydrogel.

**Figure 1:**
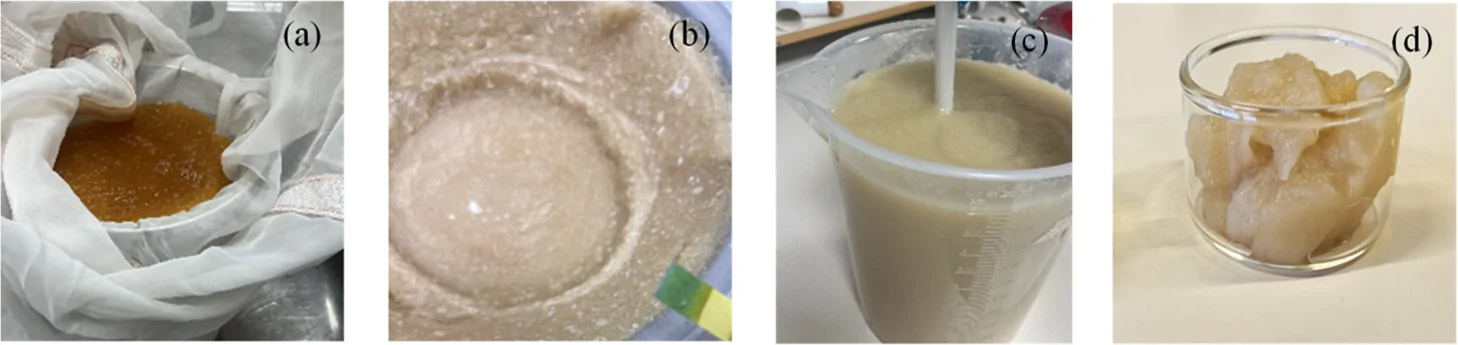
Images of the alkali insoluble material obtained by heat treatment of fungal biomass in an autoclave (a), after washing with water to neutral pH (b), after addition of lactic acid (c), and the concentrated hydrogel (5.5 wt %) (d).

#### 3.1.1 Effect of Vanillin on Mechanical Properties of Monofilaments

In a preliminary study, the hydrogel at 5.5 wt % was identified to be the best concentration for spinning monofilaments (data not shown). To conduct the filament spinning, a high-pressure syringe pump was employed which provided a uniform spinning rate and enabled the use of a needle with a diameter of 0.8 mm. This adjustment facilitated the formation of thin and uniform filaments. Using this setup, monofilaments were produced with a tenacity of 5.7 cN/tex and an elongation at break of 11%.

Vanillin is a well-known cross-linking agent for chitosan. The main mechanism of chitosan–vanillin cross-linking is Schiff-base formation, where the primary amine groups (−NH_2_) of chitosan react with the aldehyde group (−CHO) of vanillin to form imine bonds (−C=N−), creating covalent cross-links between chitosan chains.^18^ Tomadoni et al.^19^ reported that higher drying temperatures can enhance intermolecular interactions, thereby increasing the reaction between chitosan and vanillin in chitosan films. Therefore, before investigating the effect of varying vanillin concentrations as a cross-linking agent on the properties of the monofilaments, the influence of drying conditions was examined. For this purpose, vanillin was incorporated at 30 wt % concentration into the hydrogel.

Both oven drying and oven heat treatment produced brittle fibers, in contrast to the more flexible filaments obtained at room temperature. Oven drying may cause rapid water loss and stronger intermolecular interactions, in polysaccharides such as cellulose, which could reduce chain mobility and introduce internal stresses, leading to more brittle fibers. In contrast, slow room-temperature drying or drying at higher humidity levels in the oven would most likely help preserve flexibility of the material.^20^ This was consistent with the higher elongation at break measured for filaments dried at room temperature compared with those exposed to 60 °C heating (Figure 2). Furthermore, no significant improvement in tenacity or initial modulus was observed for the heat-treated samples relative to room-temperature-dried filaments. Consequently, room-temperature drying was identified as the most suitable method for preserving filament properties.

**Figure 2:**
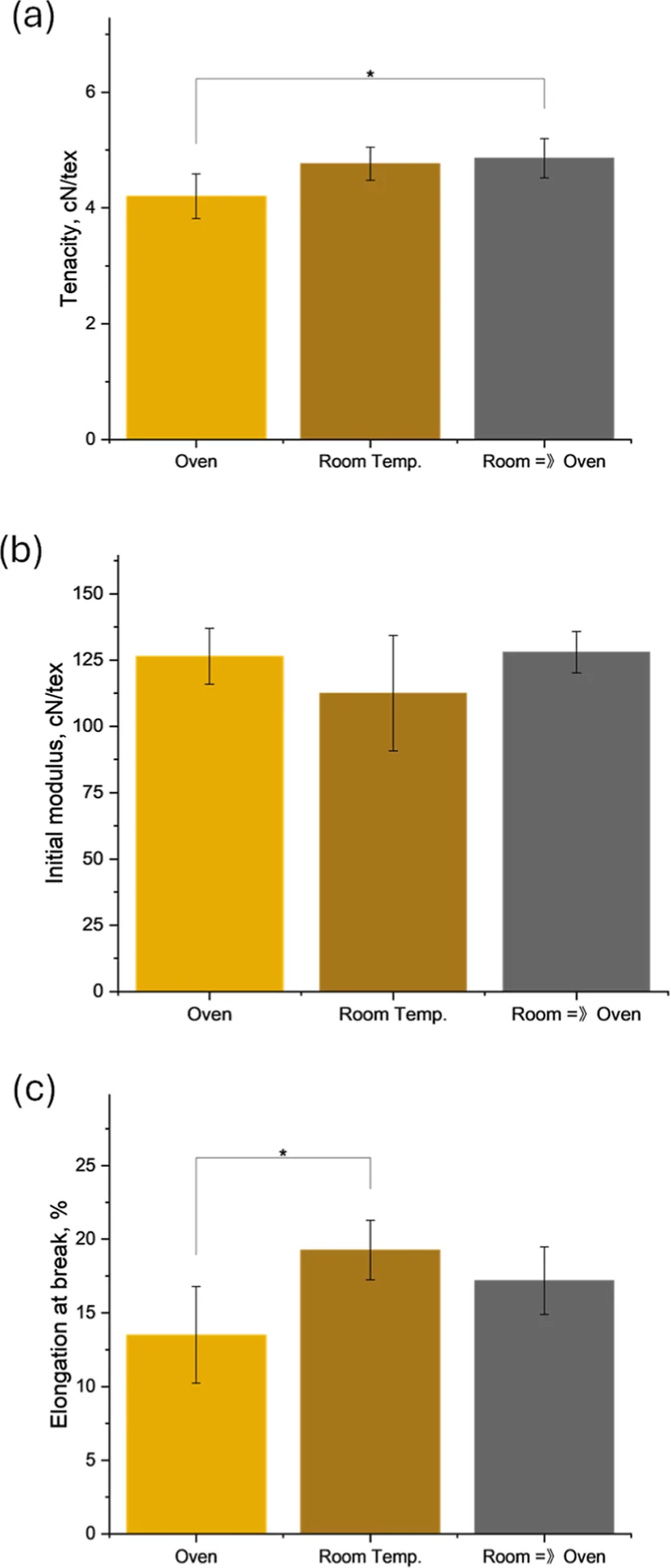
Tenacity (a), initial modulus (b), and elongation at break (c) of filaments made using fungal hydrogel containing 30 wt % vanillin under different heat treatment condition. The vanillin concentration is the percentage of vanillin relative to the dry weight of the hydrogel. The monofilaments with a linear density of 14 tex were spun using a needle with diameter of 0.8 mm and length of 40 mm. Data are presented as mean ± standard deviation (*n* = 5). Asterisks* indicate statistically differences between the indicated groups based on Tukey’s HSD test (*p* ≤ 0.05).

Integration of vanillin as a cross-linking agent into hydrogel at concentrations of 20, 40, 60, 80, and 100 wt % led to slight increases in the tenacity of the resulting monofilaments by 12.3%, 0%, 10.5%, 7.0%, and 5.3%, respectively (Table 1), compared to the non-cross-linked sample (0% vanillin). However, statistical analysis confirmed that these increases were not significant (*p* > 0.05). Similarly, no statistically significant differences were observed in breaking force or elongation at break among the samples (*p* > 0.05), although significant differences in initial modulus were observed between some samples (Table 1). This outcome can be attributed to the molecular structure of vanillin, which possesses only a single aldehyde group capable of forming one Schiff-base with chitosan.^16^ Consequently, vanillin is limited in its ability to establish a dense cross-linked network, thereby constraining improvements in mechanical strength. Additionally, in Schiff-base formation cross-linking, the imine bond is reversible and unstable which also limits the resulting improvement in mechanical strength.^21^

**Table 1:** Tensile Properties of Monofilaments Containing Different Vanillin Concentrations^b^

	tensile properties				
vanillin concentration, wt %	linear density, tex	breaking force cN	tenacity, cN/tex	initial modulus^a^, cN/tex	elongation at break, %
0	8.8	50.1 ± 4.9	5.7 ± 0.6	327.2 ± 19.3a	11.0 ± 3.2
20	8.5	53.9 ± 2.6	6.3 ± 0.3	317,8 ± 12.4a	11.9 ± 2.3
40	9.4	52.8 ± 7.9	5.6 ± 0.8	307.9 ± 23.5ab	11.1 ± 4.2
60	8.9	55.8 ± 4.5	6.3 ± 0.5	260.1 ± 9.4bc	14.8 ± 2.5
80	9.4	56.6 ± 6.1	6.0 ± 0.6	209.3 ± 42.9c	15.3 ± 3.3
100	8.6	51.7 ± 5.1	6.0 ± 0.6	266.6 ± 16.9d	12.5 ± 2.1

a For initial modulus, values sharing the same letter are not significantly different according to Tukey–Kramer’s HSD test (*p* < 0.05).

b The monofilaments were spun using a needle with a diameter of 0.8 mm and length of 40 mm. The vanillin concentration is the percentage of vanillin relative to the dry weight of the hydrogel. All values are reported as mean ± standard deviation (*n* = 5)

#### 3.1.2 Assessment of Vanillin-Induced Schiff-Base Formation by FTIR and Swelling Behavior

To further understand the effect of vanillin on the fungal hydrogel, two complementary characterization methods, FTIR spectroscopy and swelling behavior, were used to assess vanillin-induced chemical interactions as illustrated in Figure 3. FTIR analysis was performed on monofilaments containing vanillin at different concentrations and compared with vanillin-free monofilaments. The full FTIR spectra in the range of 4000–400 cm^–1^ are shown in Figure 3a. As shown in Figure 3b, spectral changes associated with vanillin incorporation and Schiff-base formation were observed in the region of 1200–1700 cm^–1^. The imine band shifted from 1643 cm^–1^ to 1661.8 cm^–1^ with increasing vanillin concentration, accompanied by a rise in intensity, which indicates the formation of Schiff-base.^22^ In addition, the amide II peak (1515.8–1536 cm^–1^) intensified in the sample containing 100% vanillin, while the N–H bending vibration of secondary amines shifted from 1564 cm^–1^ (0% vanillin) to 1587 cm^–1^ (100% vanillin). These spectral changes are consistent with the participation of amine groups in Schiff-base formation. However, Marin et al.^23^ showed that quantitative determination of imine formation from FTIR peak intensities was not reliable for vanillin–chitosan systems, since the C=N/N–H absorbance ratio did not reliably differentiate samples with different vanillin/chitosan ratios.

**Figure 3:**
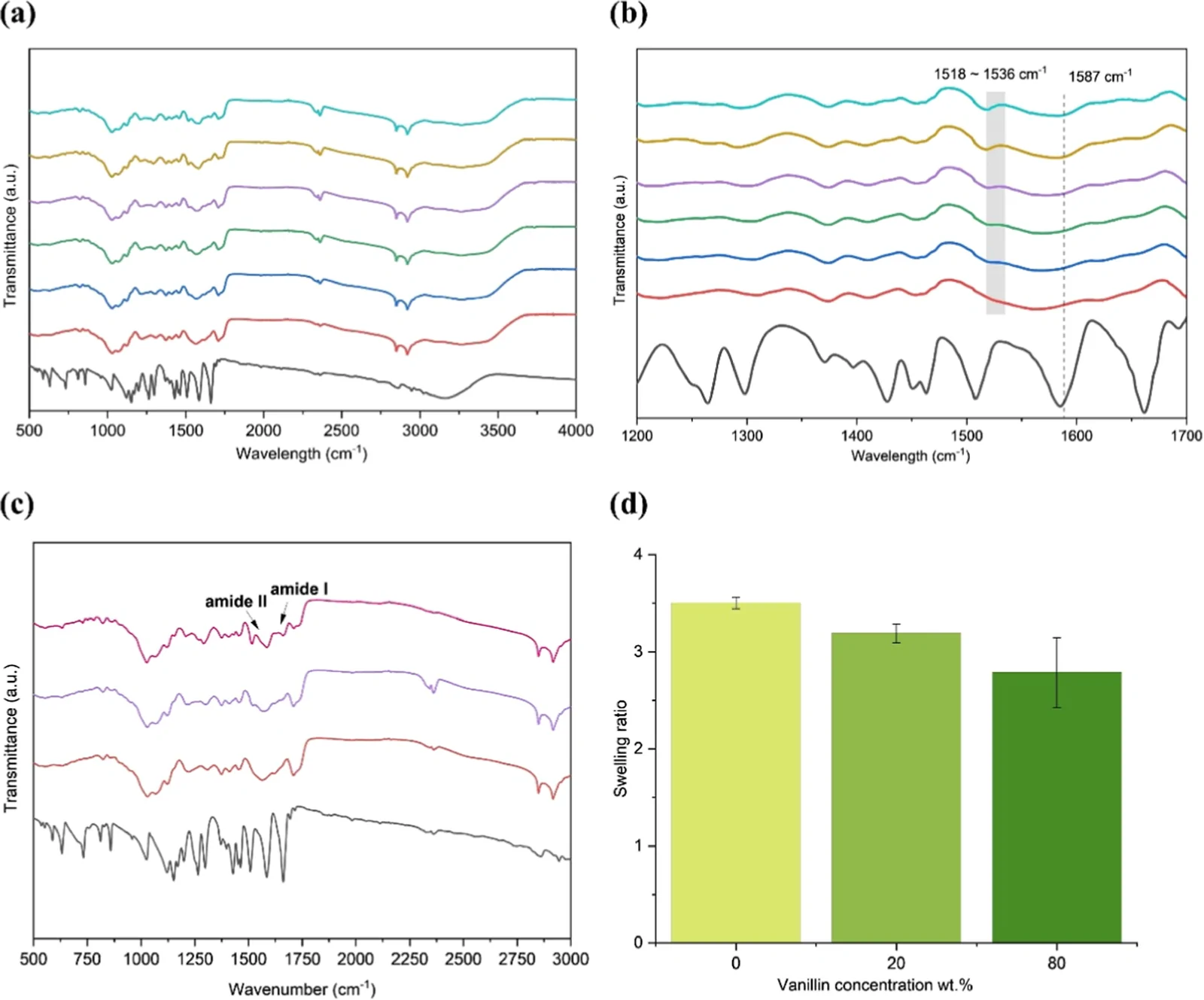
(a) FT-IR Spectra of monofilaments containing varying vanillin concentrations (0 (red), 20 (dark blue), 40 (green), 60 (purple), 80 (yellow) and 100 (light blue) wt %) and Pure Vanillin (black), with (b) FTIR spectra was expanded from 1200 to 1700 cm^–1^ enhanced for distinguishing key peaks including the Schiff-base peak. The amide I and amide II bands were identified in the FTIR spectra: (c) FTIR spectra of the monofilament without vanillin (red), monofilament with 60 wt % vanillin before (purple) and after heat treatment at 60 °C for 1 h (pink), and pure vanillin (black). The amide band I, and II are located at 1658 cm^–1^, 1580 cm^–1^ respectively; and (d) swelling ratio of monofilaments containing various vanillin concentrations. Error bars in the graph d represent standard deviation , and all data are presented as mean ± SD (*n* = 3).

Notably, Vanillin contains only one reactive aldehyde group (−CHO), enabling the formation of a single Schiff-base (−C=N−) with chitosan’s primary amines.^21^ Consequently, under ambient conditions, the extent of Schiff-base formation and resulting network modification remained limited, leading to only minor chemical modifications and subtle differences between spectra of vanillin-free and vanillin-containing monofilaments.

To promote Schiff-base formation, a postspinning thermal treatment (60% vanillin, 60 °C, 1 h) was applied. As shown in Figure 3c, the heat-treated monofilament exhibited a pronounced peak at 1617.5 cm^–1^, corresponding to C=N stretching vibrations of Schiff-base, supporting increased Schiff-base formation upon thermal processing. Comparison with spectra of vanillin-free monofilaments (VA0) revealed peak shifts and intensity changes at 1380 cm^–1^ (CH bending/CH_3_ deformation), 1154 cm^–1^ (C–O–C symmetric stretching), 1060 cm^–1^ (C–O stretching of C3–OH), and 1023 cm^–1^ (C–O stretching of C6–OH).^24^ These observations support vanillin-induced Schiff-base formation and associated hydrogen-bonding interactions. Despite the stronger spectroscopic evidence for Schiff-base formation, heat-treated filaments were too brittle to be twisted into yarns; therefore, monofilaments dried at room temperature were used for the rest of the study.

Swelling ratios were measured for selected formulations containing 0, 20, and 80% vanillin, representing the vanillin-free control and low- and high-vanillin formulations, to provide a complementary assessment of vanillin-induced changes in the monofilaments. As shown in Figure 3d, increasing the vanillin concentration reduced the swelling ratio. Compared with vanillin-free monofilament (3.50), the swelling ratio slightly decreased to 3.19 in at 20% vanillin and further decreased to 2.79 at 80% vanillin. The lower swelling ratios observed in the tested vanillin-containing monofilaments indicate reduced water uptake, consistent with increased restriction of the polymer matrix resulting from vanillin-induced reaction. However, these selected concentrations do not establish a complete concentration-dependent relationship. Together, the FTIR and swelling results provided evidence that vanillin acts as a cross-linker for chitosan within the fungal hydrogel, primarily through Schiff-base formation and hydrogen bonding.

#### 3.1.3 Effect of Glycerol as Plasticizer on Properties of Monofilaments

Plasticizers such as glycerol are widely employed to improve the flexibility, processability, and mechanical performance of biopolymer-based materials such as films, including those derived from chitosan and other polysaccharides. Glycerol acts primarily by disrupting intermolecular hydrogen bonding, thereby increasing free volume and enhancing polymer chain mobility.^25,26^ Beyond films, glycerol has also been utilized in the textiles as a lubricant and softener for both biobased and synthetic fibers and yarns, improving flexibility and facilitating handling.^27–29^ Furthermore, glycerol serves multiple roles in textile processing and finishing, including as a wetting agent in dyeing and printing, a humectant to retain fabric moisture, an antistatic additive, and a finishing agent that enhances softness and tactile comfort.^30,31^

Given these established functions, we previously investigated the effect of glycerol post-treatment on the properties of wet-spun fungal monofilaments. This treatment involved immersing dried filaments in an aqueous glycerol solution followed by redrying, which resulted in increased elongation at break.^9^ When a similar post-treatment was applied to dry gel-spun fungal filaments in preliminary experiments, their flexibility improved; however, accurately controlling the amount of glycerol absorbed by each monofilament proved challenging. Moreover, rewetting the filaments complicated their handling and processing. To overcome these limitations, in the present study, glycerol was incorporated directly into the spinning dope. This approach ensured a more uniform distribution of glycerol along the filament length and eliminated the need for rewetting during post-treatment.

As shown in Table 1, monofilaments produced using a 0.8 mm needle exhibited low linear densities, corresponding to finer filaments. However, these finer filaments were more prone to tangling and were difficult to handle, especially when producing longer lengths of plied yarn during twisting. Therefore, a 1.2 mm needle was used in the subsequent experiments to investigate the effect of glycerol addition on the properties of the monofilaments.

Table 2 summarizes the tensile properties of fungal monofilaments containing different glycerol concentrations at a fixed vanillin content of 20 wt %. Increasing glycerol concentration led to an overall increase in elongation at break, accompanied by reductions in tenacity and initial modulus. At the same time, the linear density progressively increased from 48 tex without glycerol to 68 tex at 20 wt % glycerol. Since tenacity and initial modulus are normalized by linear density, this increase needs to be considered when interpreting the reduction in these normalized properties. To distinguish the linear density effect from the plasticizing effect of glycerol, absolute breaking force was also evaluated (Table 2). Breaking force showed an overall decrease with increasing glycerol concentration, demonstrating that the reduction in tenacity cannot be attributed solely to the increase in filament linear density. At 20 wt % glycerol, a partial recovery in breaking force was observed; however, the value remained substantially below that of the glycerol-free filament.

**Table 2:** Tensile Properties of Monofilaments Containing Varying Glycerol Concentrations, Prepared with (20 wt %) and Without Vanillin^b^

		tensile properties				
glycerol concentration (wt %)	vanillin concentration^a^, %	linear density, tex	breaking force, cN	tenacity, cN/tex	initial modulus, cN/tex	elongation at break, %
0	20	48	167.5 ± 18.8	3.5 ± 0.4	91.5 ± 33.4	12.8 ± 5.6
5	20	52	102.6 ± 24.5	2.0 ± 0.5	8.7 ± 4.0	29.0 ± 2.6
10	20	59	64.9 ± 7.4	1.1 ± 0.1	5.3 ± 0.2	18.9 ± 2.4
15	20	63	63.6 ± 12.0	1.0 ± 0.2	4.6 ± 0.6	19.3 ± 3.4
20	20	68	86.2 ± 12.5	1.3 ± 0.2	6.2 ± 1.9	23.0 ± 3.6
0	-	36	202.6 ± 15.2	5.7 ± 0.4	115.8 ± 9.0	21.4 ± 2.7
5	-	56	162.4 ± 9.6	2.9 ± 0.2	11.0 ± 0.6	25.9 ± 1.8
20	-	56	94.9 ± 6.8	1.7 ± 0.1	9.7 ± 0.0	16.1 ± 1.3

a Note: vanillin concentration; “-“indicates filaments spun without vanillin.

b All samples were spun using a needle with dimensions of 1.2 mm × 50 mm. Values are reported as mean ± standard deviation (*n* = 5 for vanillin-containing samples and *n* = 10 for vanillin-free sample).

Among the glycerol-containing filaments, those containing 5 wt % glycerol exhibited the highest tenacity (2.0 ± 0.5 cN/tex) and initial modulus (8.7 ± 4.0 cN/tex). Statistical analysis confirmed that glycerol concentration had a significant effect (*p* ≤ 0.05) on elongation at break, tenacity, and initial modulus. The observed decrease in tenacity and increase in elongation are attributed to the plasticizing effect of glycerol, which disrupts intermolecular interactions within the filament structure, increases free volume and enhances polymer chain mobility in the cell wall material. Similar plasticization trends have been reported for chitosan-based films treated with glycerol.^19,32^

To further investigate the role of glycerol in filament flexibility, additional filaments were prepared without vanillin at selected glycerol concentrations (0, 5, and 20 wt %), and their tensile properties are presented in Table 2. These concentrations were selected to represent the unplasticized condition, a low glycerol concentration, and the highest glycerol concentration investigated, respectively. The vanillin-free series was used to determine whether sufficient plasticization and flexibility could also be achieved in the absence of vanillin and was therefore not intended as a complete concentration-matched comparison with the vanillin-containing formulations.

The vanillin-free filaments showed a similar response to glycerol addition, with an overall reduction in absolute breaking force. This indicates that the reduction in load-bearing capacity with increasing glycerol content also occurs in the absence of vanillin, further supporting the plasticizing effect of glycerol. At 0 wt % glycerol, vanillin-free filaments exhibited substantially higher tenacity (5.7 ± 0.4 cN/tex) and initial modulus (115.8 ± 9.0 cN/tex) than filaments containing glycerol, indicating a stiffer and mechanically stronger structure. Upon glycerol addition, the vanillin-free filaments similarly exhibited reduced tenacity and modulus together with changes in elongation at break. At comparable glycerol concentrations, vanillin-free filaments generally retained higher tenacity than the corresponding vanillin-containing filaments. However, differences in linear density between the formulations complicate direct comparison of the normalized tensile properties. Importantly, the breaking-force data showed an overall reduction in absolute load-bearing capacity with glycerol addition in both vanillin-containing and vanillin-free filaments. Thus, the effect of glycerol on filament mechanical performance is not solely associated with the presence of vanillin.

At higher linear densities, vanillin-free filaments exhibited higher tenacity and modulus than vanillin-containing filaments (Table 2), in contrast to the comparable performance observed for filaments with vanillin and without glycerol at lower linear densities (Table 1). Differences in filament fineness should therefore be considered when comparing the different formulations.

Beyond these processing-related effects, the hygroscopic nature of glycerol also makes the moisture condition of the resulting filaments an important consideration. During handling, excessive drying increased filament brittleness, whereas high moisture exposure reduced filament stability. These qualitative observations highlight the need for further studies under controlled relative humidity conditions to evaluate the mechanical and dimensional stability of the filaments.

To assess the mechanical performance of the fungal filaments in the context of existing textiles, their properties were compared with those of chitosan-based and established textile fibers. Kuznik et al.^33^ reported that undrawn pure-chitosan monofilaments with linear densities of approximately 13–45 tex exhibited tenacities of 4.41–6.09 cN/tex and elongations at break of approximately 13–33%, partially overlapping with the ranges obtained for the present fungal filaments (36–68 tex, 1.0–5.7 cN/tex, and 12.8–29. %, respectively). Nechyporchuk et al.^34^ further showed that filament drawing substantially improved the tenacity of wet-spun chitosan fibers through increased structural orientation, suggesting a potential route for further optimization of the fungal filaments.

A broader comparison using an Ashby plot (Figure 4) showed that, although the elongation at break of the fungal filaments falls within the range of some textile fibers, their specific strength remains lower than that of many natural fibers including cellulose fibers. Further improvements in filament fineness, filament drawing, and cross-linking chemistry may therefore help narrow this performance gap. Ashby’s material selection charts, developed by Prof. Mike Ashby, are a widely used approach for comparing materials based on their properties^35,36^

**Figure 4:**
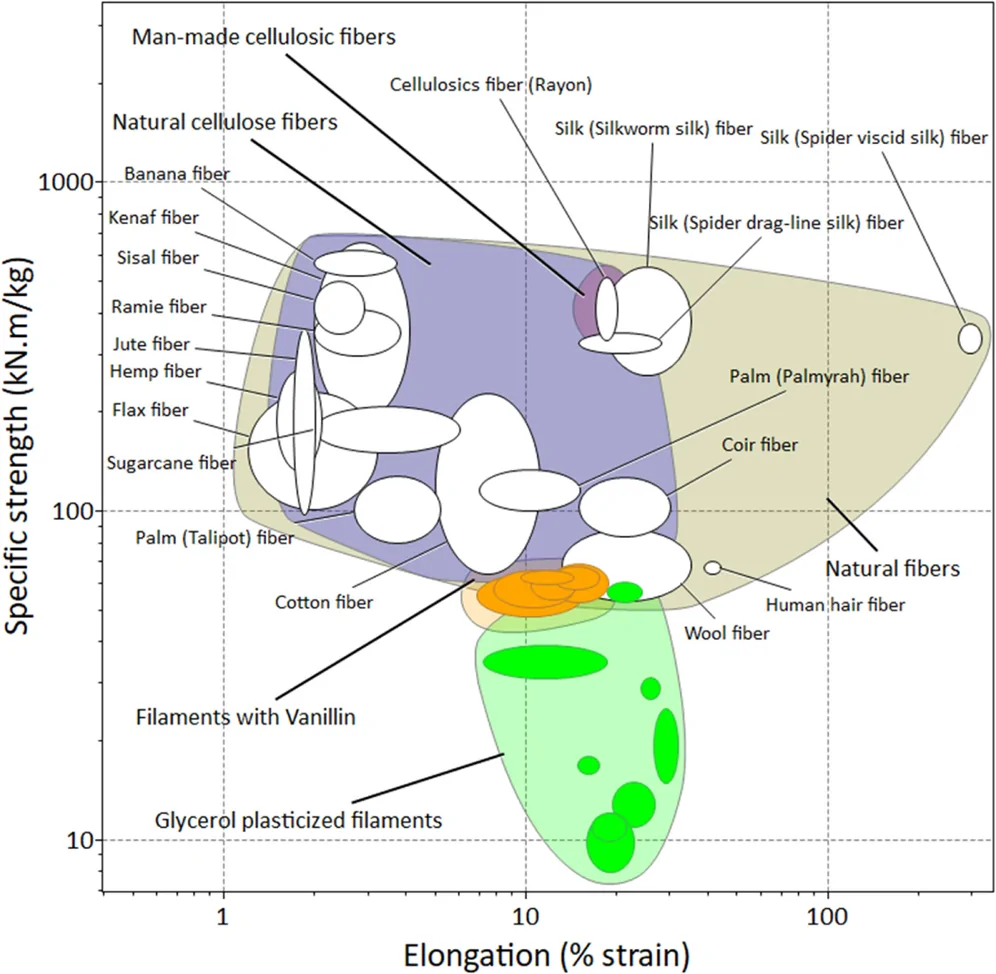
Ashby material property chart comparing the mechanical performance of the fungal-derived filaments with established textile fibers. Specific strength is plotted against elongation at break. Orange symbols represent vanillin-containing filaments and green symbols represent glycerol-plasticized filaments. (Plotted using Granta EduPack 2025 R2, (version 21.2.0, Ansys Inc., USA).

#### 3.1.4 Morphology of the Monofilaments

Morphology of the monofilaments was examined using SEM from both the surface and cross-sectional perspectives (Figure 5). The monofilament without vanillin showed a well-aligned and relatively smooth surface, indicating parallel organization of the fungal microfibers along the filament axis, consistent with previous reports on fungal filaments produced by wet-spinning and dry-gel spinning.^9,11^ In contrast, the monofilament containing 20 wt % vanillin exhibited a rougher and less uniform surface, suggesting that the presence of vanillin interfered with the microfiber alignment during filament formation.

**Figure 5:**
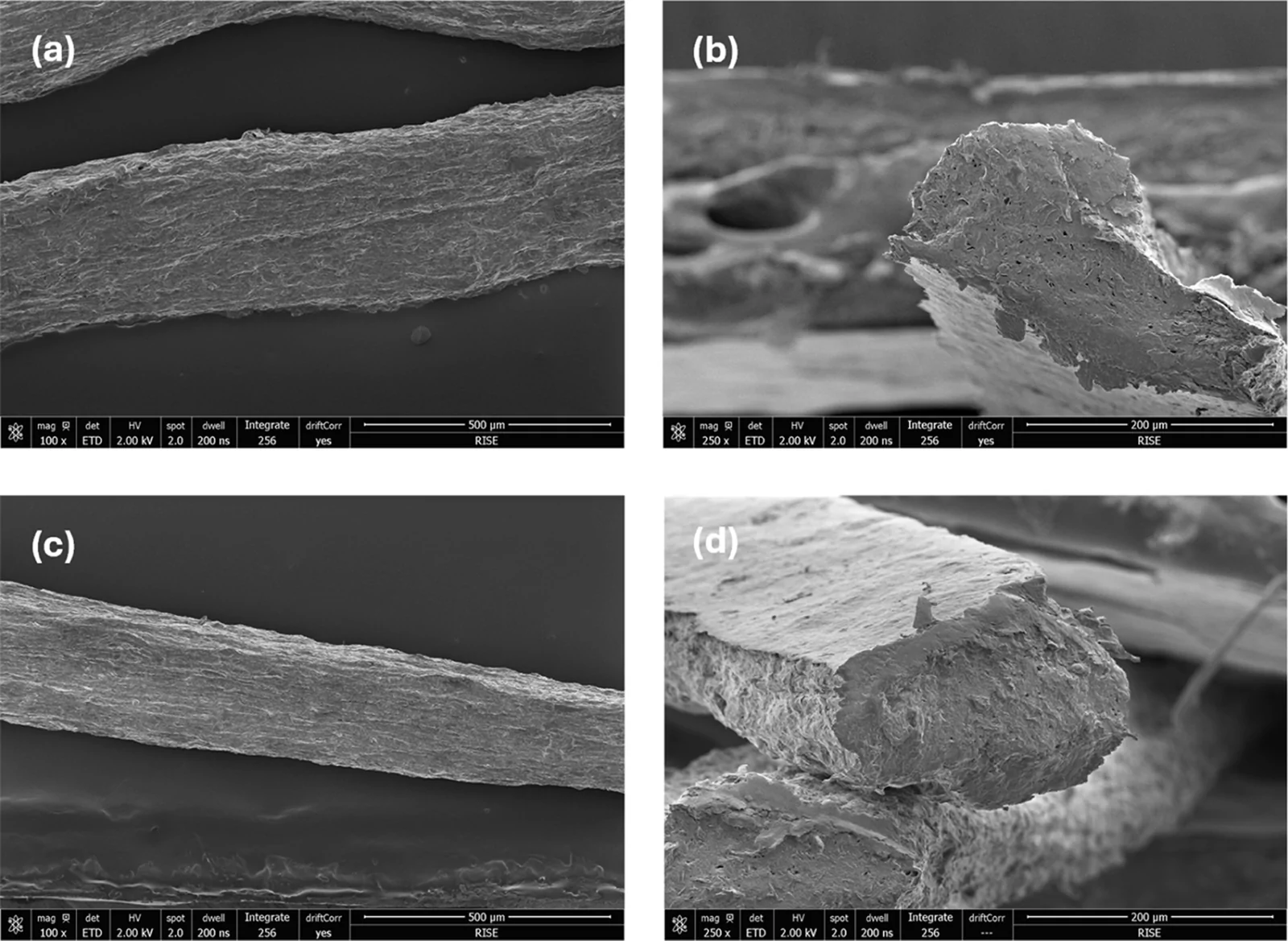
Surface and cross-section images of monofilaments at 100× and 250× magnification, respectively: (a,b) containing 20 and 20 wt % vanillin; (c,d) containing 20 wt % glycerol and no vanillin.

The cross-sectional morphology further supported this trend: the monofilament without vanillin showed a more cohesive and compact internal structure, whereas the filament containing vanillin displayed a more irregular and fragmented morphology. It is important to note that these observations may be influenced to some extent by the sample preparation and cross-sectioning procedure used prior to SEM analysis. Therefore, the observed morphology should be interpreted as a comparative assessment between samples prepared under the same experimental conditions, rather than as a definitive representation of the absolute internal structure. This structural disruption is consistent with the tensile testing results, where the glycerol-only fibers demonstrated slightly higher tenacity compared with the vanillin-containing fibers (Table 2). Despite these differences, both samples consistently exhibited an elliptical cross-section.

The structural anisotropy and molecular alignment of the fungal filaments were evaluated using polarized light microscopy (PLM) in transmission mode under crossed polarizers. When oriented at a 45° relative to the polarizer transmission directions, the neat fungal filament (0 wt % glycerol, 0 wt% vanillin) exhibited strong, fibrillar birefringence (Figure 6a). This pronounced optical retardation reflects an ordered, anisotropic network of crystalline microfibrils aligned parallel to the longitudinal fiber axis. Upon rotating the filament to 0° (parallel to the polarizer axis), the transmitted light intensity dropped sharply to complete optical extinction (Figure 6c). This uniform extinction behavior confirms a high degree of uniaxial molecular orientation along the fiber axis with minimal disoriented crystalline domains.

**Figure 6:**
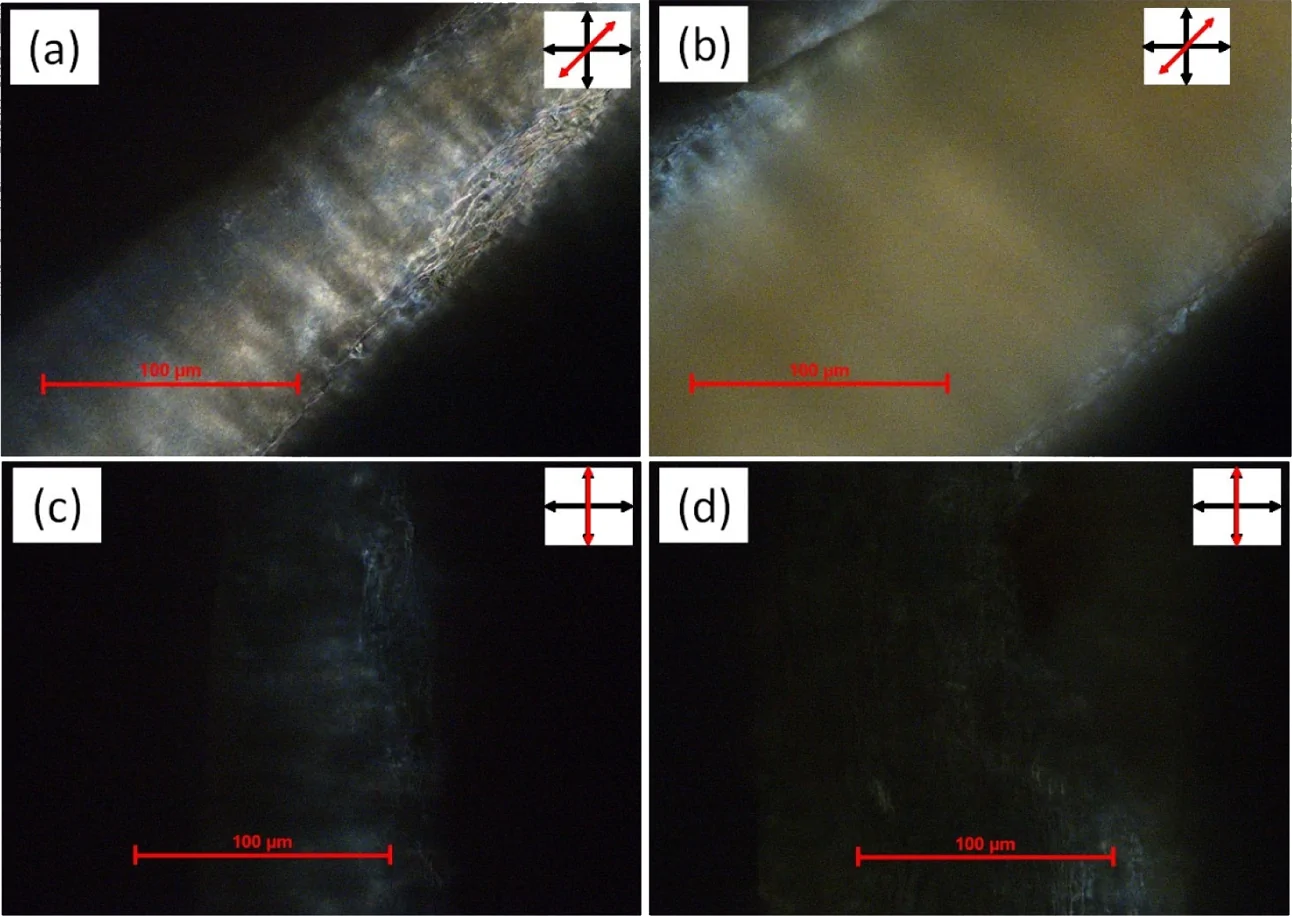
Polarized light microscopy micrographs of fungal monofilaments captured in transmission mode under crossed polarizers (scale bars = 100 μm). (a) Neat fungal filament (0 wt % glycerol, 0 wt% vanillin) oriented at 45° relative to the polarizer axis. (b) Plasticized fungal filament (20 wt % glycerol, 0 wt% vanillin) oriented at 45°. (c) Neat fungal filament rotated to 0° (optical extinction). (d) Plasticized fungal filament rotated to 0° (optical extinction). Inset arrows denote the filament longitudinal axis (red) relative to the polarizer and analyzer transmission axes (black crosshairs). Scale bar = 100 μm.

The incorporation of 20 wt % glycerol altered the optical transmission characteristics while maintaining the overarching macro-axial alignment of the monofilament. At the 45° diagonal position, the plasticized filament exhibited a visibly smoother, attenuated birefringence with reduced optical retardation compared to the neat sample (Figure 6b). This optical damping confirms effective plasticization, wherein glycerol molecules penetrate the biopolymeric network, interrupt intermolecular hydrogen bonds, and expand the amorphous free volume. Despite this drop in local crystalline intensity, the plasticized filament retained sharp, complete optical extinction upon rotation to 0° (Figure 6d). This confirms that the shear-induced longitudinal chain orientation established during spinning remained intact after plasticizer addition. These optical findings align with the tensile behavior, where plasticization lowered tenacity while providing the ductility necessary for continuous yarn twisting and knitting. Although these observations provide qualitative evidence of axial orientation within the fungal filaments, PLM does not provide a quantitative measure of individual microfiber alignment. Similar polarized-light approaches have previously been used to assess structural orientation in spun cellulose nanofibril (CNF) filaments, where angular changes in birefringence and extinction behavior were associated with preferential fibril alignment.^37,38^

### 3.2 Twisting of Fungal Monofilaments

Initial twisting trials using monofilaments without glycerol were feasible but resulted in frequent breakage during the twisting due to their brittleness. The incorporation of glycerol markedly improved twisting performance, substantially reducing filament breakage during processing. This improvement can be attributed to the three hydroxyl groups in glycerol, which form hydrogen bonds with the hydrophilic polymer chains and increase intermolecular spacing by disrupting internal hydrogen bonding.^39^ As a result, the addition of glycerol reduced the brittleness of the fungal monofilaments and imparted the desired stretchability and flexibility. A comparable effect has been reported for paper yarns, where glycerol significantly enhanced yarn processability.^27^

Yarn stability was further dependent on twist count and filament number. When the twist exceeded 24 TPM or when yarns contained more than six monofilaments, filament breakage occurred during processing. These observations indicate that stable plied yarns are best achieved using 4–5 monofilaments with twist counts below 24 TPM. In addition, yarns composed of three monofilaments containing vanillin without glycerol, as well as those containing a low glycerol content (5 wt %), could be knitted but exhibited considerable filament breakage due to insufficient flexibility. In contrast, yarns prepared from monofilaments containing higher glycerol contents (15–20 wt %) showed substantially improved knittability, highlighting the critical role of glycerol concentration in enabling yarn processability. Figure 7a,b presents microscopic images of twisted multifilament yarns, confirming the formation of stable twisted yarns composed of three monofilaments.

**Figure 7:**
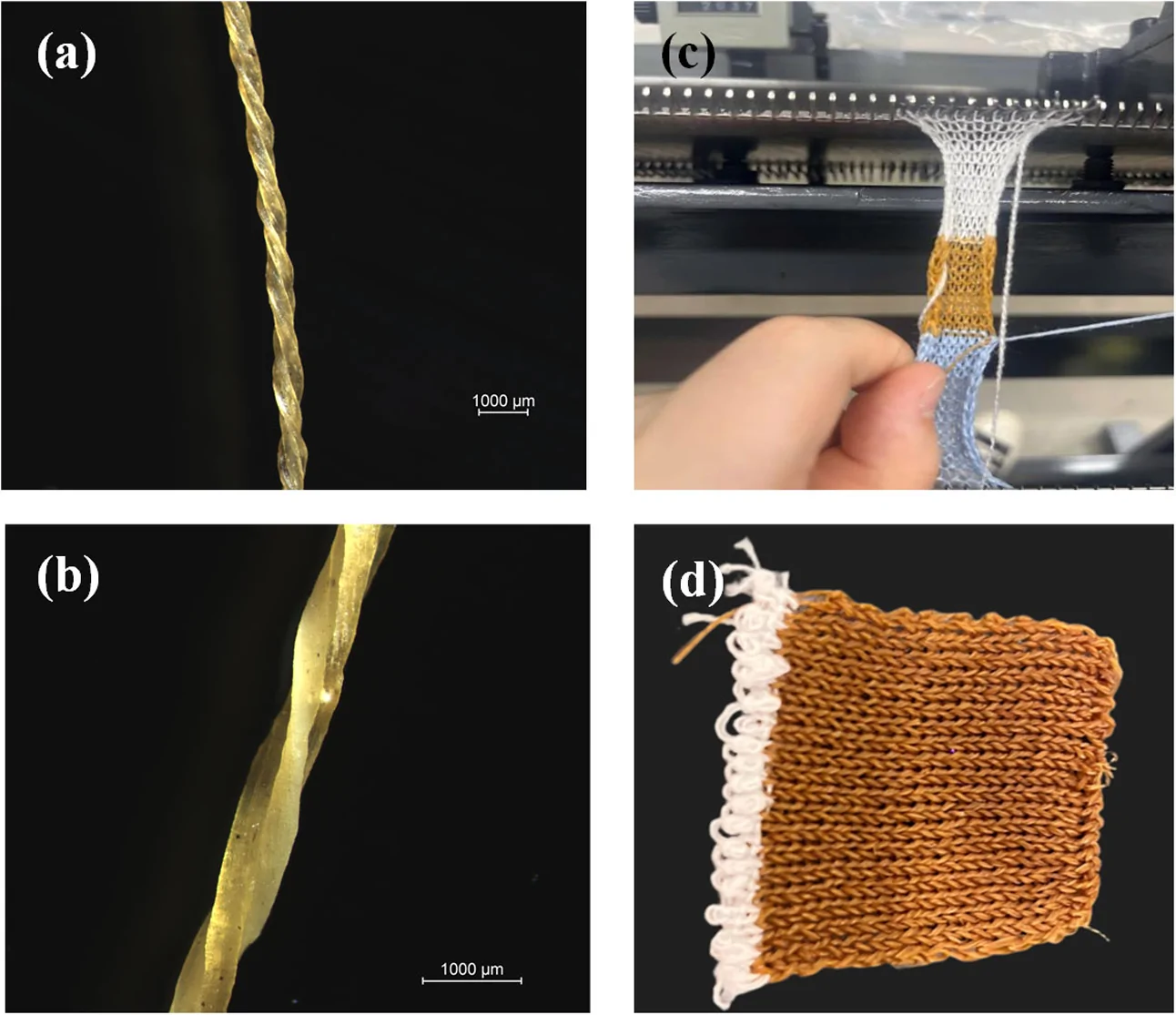
Microscopic images of twisted multifilament yarns made using three monofilaments containing (a) no vanillin and 5% glycerol and (b) 20% vanillin and 20% glycerol; (c) knitting of yarn containing 20% vanillin and 5% glycerol; and (d), knitted fabric produced from yarn containing no vanillin and 20% glycerol.

### 3.3 Knitting

This study presents the first demonstration of fungal filaments being processed through a conventional textile operation, namely knitting as a key step in assessing their suitability as textile materials. Initial trials with both vanillin-free and vanillin-cross-linked monofilaments lacking plasticizer revealed that the filaments were too brittle to undergo knitting. Incorporation of glycerol as a plasticizer improved filament flexibility and processability. Knitting was technically possible at 5 wt % glycerol; however, considerable filament breakage occurred during processing and was visible in the resulting knitted structures. Increasing the glycerol concentration to 10 wt % or higher substantially reduced filament breakage and enabled more reliable knitting (Figure 7c). Notably, even vanillin-free filaments could be knitted when plasticized with 20% wt % glycerol (Figure 7d), highlighting the crucial role of glycerol in imparting flexibility and enabling textile processing. Enhanced knittability of paper-yarn due to treatment with glycerol has similarly been reported.^27^

Knittability was further influenced by both filament count and glycerol concentration. Yarns composed of six monofilaments could not be knitted even at the highest glycerol concentration tested, while those containing four or five monofilaments were successfully knitted at lower glycerol levels. These results demonstrate that both filament number and glycerol concentration play crucial roles in determining the knittability of fungal multifilaments.

Figure 8 presents microscopic images of knitted fabrics produced from twisted fungal monofilament yarns. Although knitting was possible for yarns composed of four twisted monofilaments, containing relatively low glycerol concentration (5 wt %) with and without vanillin, visible monofilament breakage can be seen on the fabric surface (Figure 8a). This damage is due to the increased brittleness of the fungal filaments at low glycerol contents, as well as the higher number of monofilaments in the twisted yarns. In contrast, knitted fabrics produced from twisted filaments containing 10% or more glycerol exhibited significantly fewer fiber breaks, confirming the enhanced flexibility imparted by glycerol (Figure 8b). Notably, the specimen made from yarns containing no vanillin demonstrated excellent processability, showing no evidence of filament breakage on the fabric surface. Moreover, even the closed loops in the knitted structure were formed entirely from fungal filaments, indicating that the yarn possessed sufficient strength and flexibility during the knitting process. SEM images of fabrics made filaments containing 20% glycerol, with and without vanillin (Figure 8c,d) further confirmed the formation of well-organized knitted structures.

**Figure 8:**
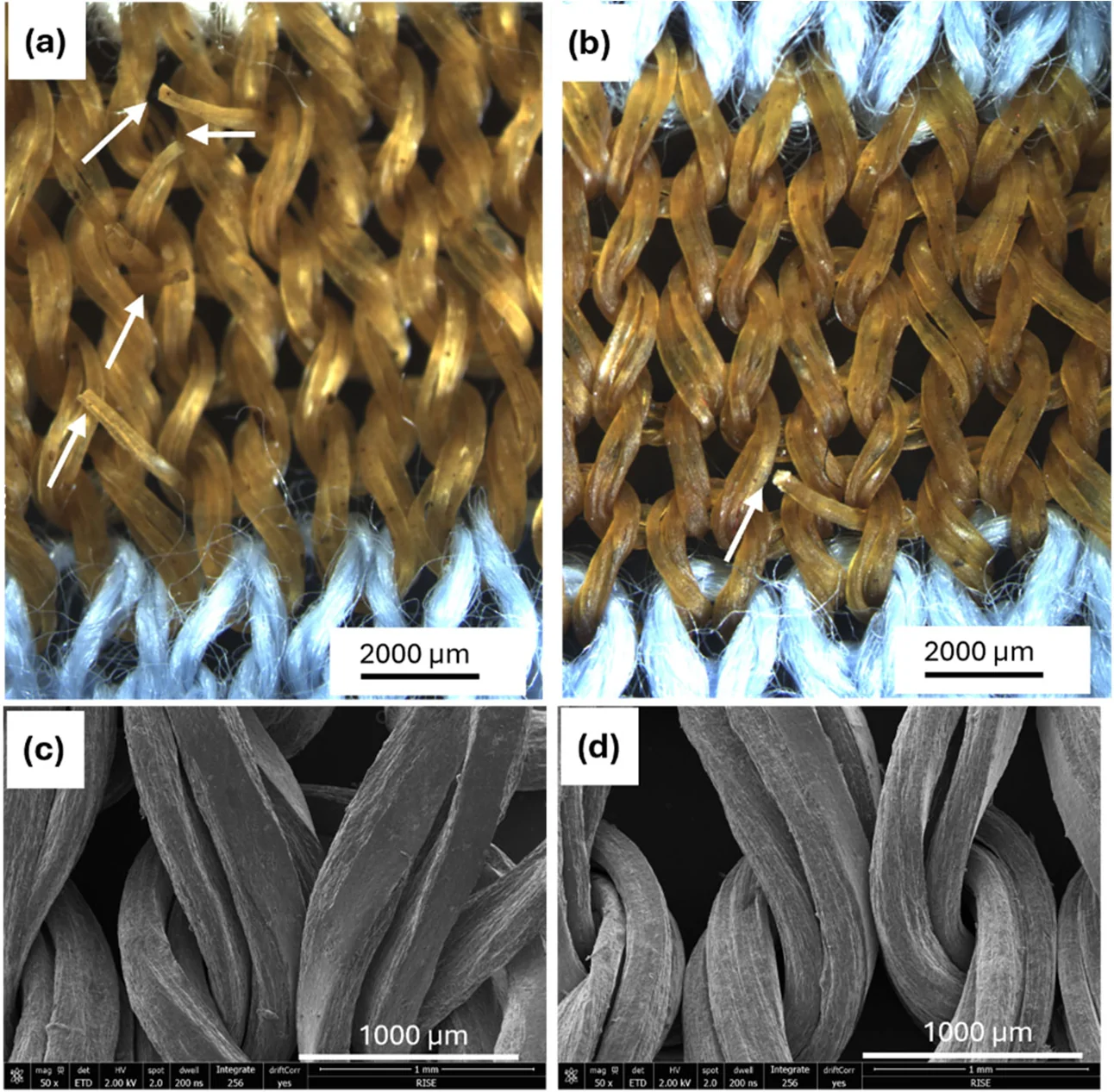
Optical microscopic images of knitted fabrics prepared from yarns containing (a) 0 wt % vanillin and 5 wt % glycerol, and (b) 20 wt % vanillin and 15 wt % glycerol. Arrows in the insets indicate breakage of individual monofilaments. SEM images of knitted fabrics prepared from yarns containing (c) 20 wt % vanillin and 20 wt % glycerol, and (d) 0 wt % vanillin and 20 wt % glycerol. Yarns in panel (a) consist of four monofilaments, whereas those in panels (b–d) consist of three monofilaments.

## 4 Conclusions

This study demonstrates the production of textile quality filaments from cell wall materials of *R. delemar* grown on bread waste using dry-gel spinning. Vanillin acted as a cross-linker, confirmed by FTIR and swelling tests, but due to its single aldehyde group, it showed limited effect on improving the tenacity. Filament fineness is a critical parameter in hydrogel spinning, therefore further investigation of potential cross-linking agents should systematically account for fiber fineness and processing conditions to properly assess their effectiveness. Glycerol served as an effective plasticizer, enhancing filament flexibility and enabling twisting and knitting, although at the expense of reduced tenacity.

Importantly, this work provides the first proof-of-concept knitted fabrics composed entirely of fungal-derived filaments, processed on a conventional knitting machine without modification. These findings highlight the strong potential of fungal filaments as sustainable textile materials while emphasizing the need to further optimize the balance between strength and flexibility, as well as to evaluate their mechanical and dimensional stability under varying humidity conditions. Incorporating alternative cross-linking agents containing multiple aldehyde groups may offer a pathway to improve the mechanical performance of fungal filaments.

## References

[1] Bartek L. (2025). The power of prevention and valorisation – Environmental impacts
of reducing surplus and waste of bakery products at retail. Sustain. Prod. Consum..

[2] Rahaman M. T. (2024). Green production and
consumption of textiles and apparel: Importance,
fabrication, challenges and future prospects. J Open Innov. Technol. Mark. Complex..

[3] Täuber S., Riedel S. L., Junne S. (2025). Polyhydroxyalkanoate
production from
food residues. Appl. Microbiol. Biotechnol..

[4] Uddin M. K. (2024). Polyhydroxyalkanoates,
A prospective solution in the textile industry
- A review. Polym. Degrad. Stab..

[5] Esmail A. (2025). Bacterial cellulose
production through the valorization of waste
apple pulp and stale bread. Biomass Convers.
Biorefin..

[6] Takagi A. (2025). High strength kami-ito yarns from microbial cellulose biofilms. Int. J. Biol. Macromol..

[7] Svensson S., Abdollahi M., Moghadam F. H., Kalita N. K., Hakkarainen M., Wijayarathna E. R. K. B., Mohammadkhani G., Ferreira J. A., Zamani A. (2024). Valorization of Bread Waste to Fungal-Based
Products for Medical Textile and Food Applications. ACS Sustainable Resour. Manage..

[8] Svensson S. E. (2021). Valorization of Bread Waste to a Fiber- and
Protein-Rich Fungal Biomass. Fermentation.

[9] Kanishka
B Wijayarathna E. R., Mohammadkhani G., Moghadam F. H., Berglund L., Ferreira J. A., Adolfsson K. H., Hakkarainen M., Zamani A. (2024). Tunable Fungal Monofilaments from Food Waste for Textile
Applications. Glob. Chall..

[10] Svensson S. E., Ferreira J. A., Hakkarainen M., Adolfsson K. H., Zamani A. (2021). Fungal textiles: Wet spinning of fungal microfibers
to produce monofilament yarns. Sustainable Mater.
Technol..

[11] Lindh A., Wijayarathna E. R. K. B., Ciftci G. C., Syed S., Bashir T., Kadi N., Zamani A. (2024). Dry gel spinning of
fungal hydrogels for the development of renewable yarns from food
waste. Fungal Biol. Biotechnol..

[12] Rivero S., García M. A., Pinotti A. (2010). Crosslinking capacity
of tannic acid
in plasticized chitosan films. Carbohydr. Polym..

[13] Priyadarshi R. (2018). Chitosan film incorporated
with citric acid and glycerol as an active
packaging material for extension of green chilli shelf life. Carbohydr. Polym..

[14] Wang J., Zhuang S. (2022). Chitosan-based materials:
Preparation, modification
and application. J. Cleaner Prod..

[15] Westlake J. R. (2023). Vanillin Cross-Linked Chitosan Film with Controlled
Release of Green
Tea Polyphenols for Active Food Packaging. ACS
Food Sci. Technol..

[16] Yu H. (2023). Vanillin cross-linked
chitosan/gelatin bio-polymer film with antioxidant,
water resistance and ultraviolet-proof properties. Int. J. Biol. Macromol..

[17] Svensson S. E. (2021). Fungal textiles: Wet
spinning of fungal microfibers to produce monofilament
yarns. Sustainable Mater. Technol..

[18] Xu C. (2018). Self-healing chitosan/vanillin hydrogels based on Schiff-base
bond/hydrogen
bond hybrid linkages. Polym. Test..

[19] Tomadoni B. (2019). Vanillin as a natural
cross-linking agent in chitosan-based films:
Optimizing formulation by response surface methodology. Polym. Test..

[20] Sjöstrand B. (2024). Hornification in Commercial
Chemical Pulps: Dependence on Drying
Temperature. BioResources.

[21] Hu J. (2021). Vanillin-bioglass cross-linked
3D porous chitosan scaffolds with
strong osteopromotive and antibacterial abilities for bone tissue
engineering. Carbohydr. Polym..

[22] Eghbali H. (2023). Vanillin crosslinked 3D porous chitosan hydrogel for
biomedicine
applications: Preparation and characterization. J. Mech. Behav. Biomed. Mater..

[23] Marin L. (2013). Antifungal vanillin–imino-chitosan
biodynameric films. J. Mater. Chem. B.

[24] Stroescu M. (2015). Chitosan-vanillin composites
with antimicrobial properties. Food Hydrocolloids.

[25] Sun Y. (2020). Effects of plasticizer
type and concentration on rheological, physico-mechanical
and structural properties of chitosan/zein film. Int. J. Biol. Macromol..

[26] Suwanamornlert P., Taksima T., Thanyacharoen-anukul P. (2023). Effects of
Plasticizers
on Characterization of Biodegradable Film Based on Tamarind Kernel
Polysaccharide. Int. J. Curr. Trends Sci. Technol..

[27] Syrén F., Andersson Drugge G., Peterson J., Kadi N. (2021). Enhanced Knittability
of Paper Yarn from the Swedish Forest by Using Textile Finishing Materials. Polymers.

[28] Thill S., Gebhardt R. (2022). Effect of Glycerol,
Calcium and Transglutaminase Post-Treatment
on the Properties of Regenerated Fibers from Rennet-Treated Casein
Micelles. Colloids Interfaces.

[29] Im Y. M. (2020). Effects of Glycerol on Melt Spinning of Polyacrylonitrile Copolymer
and Tetrapolymer. Fibers Polym..

[30] Leffingwell G. (1944). Textile Applications
for Glycerine. Text. Res..

[31] Ferrero F., Periolatto M. (2012). Glycerol in
comparison with ethanol in alcohol-assisted
dyeing. J. Cleaner Prod..

[32] Cissé M. (2012). Influence of the Concentrations of Chitosan
and Glycerol on Edible
Film Properties Showed by Response Surface Methodology. J. Polym. Environ..

[33] Kuznik I., Kruppke I., Cherif C. (2022). Pure Chitosan-Based
Fibers Manufactured
by a Wet Spinning Lab-Scale Process Using Ionic Liquids. Polymers.

[34] Nechyporchuk O. (2020). Wet Spinning of Chitosan
Fibers: Effect of Sodium Dodecyl Sulfate
Adsorption and Enhanced Dope Temperature. ACS
Appl. Polym. Mater..

[35] Ashby M. F., Cebon D. (1993). Materials selection
in mechanical design. J.
Phys..

[36] Shah D. (2014). Natural fibre
composites: Comprehensive Ashby-type materials selection charts. Mater. Des..

[37] Håkansson K. M.
O. (2014). Hydrodynamic
alignment and assembly of nanofibrils
resulting in strong cellulose filaments. Nat.
Commun..

[38] Mohammadi P., Toivonen M. S., Ikkala O., Wagermaier W., Linder M. B. (2017). Aligning cellulose nanofibril dispersions
for tougher
fibers. Sci. Rep..

[39] Eslami Z. (2023). A Review of the Effect of Plasticizers on the Physical
and Mechanical
Properties of Alginate-Based Films. Molecules.

